# The synergic effects of presynaptic calcium channel antagonists purified from spiders on memory elimination of glutamate-induced excitotoxicity in the rat hippocampus trisynaptic circuit

**DOI:** 10.3389/fmolb.2023.1243976

**Published:** 2023-11-30

**Authors:** Mohammad Keimasi, Kowsar Salehifard, Noushin Mirshah Jafar Esfahani, Fariba Esmaeili, Arman Farghadani, Mohammadreza Amirsadri, Mohammadjavad Keimasi, Maryam Noorbakhshnia, Majid Moradmand, Mohammad Reza Mofid

**Affiliations:** ^1^ Department of Plant and Animal Biology, Faculty of Biological Sciences and Technology, University of Isfahan, Isfahan, Iran; ^2^ Department of Physiology, School of Medicine, Isfahan University of Medical Sciences, Isfahan, Iran; ^3^ Department of Biology, Faculty of Biological Sciences, University Duisburg-Essen, Essen, Germany; ^4^ Department of Clinical Pharmacy and Pharmacy Practice, School of Pharmacy and Pharmaceutical Sciences, Isfahan University of Medical Sciences, Isfahan, Iran; ^5^ Department of Clinical Biochemistry, School of Pharmacy and Pharmaceutical Sciences, Isfahan University of Medical Sciences, Isfahan, Iran

**Keywords:** cognitive dysfunction, memory, calcium channel blockers, calcium channel Cav2.2 (N type), calcium channel Cav2.1 (P/Q type), long-term potentiation, mossy fiber, Schaffer collateral

## Abstract

The hippocampus is a complex area of the mammalian brain and is responsible for learning and memory. The trisynaptic circuit engages with explicit memory. Hippocampal neurons express two types of presynaptic voltage-gated calcium channels (VGCCs) comprising N and P/Q-types. These VGCCs play a vital role in the release of neurotransmitters from presynaptic neurons. The chief excitatory neurotransmitter at these synapses is glutamate. Glutamate has an essential function in learning and memory under normal conditions. The release of neurotransmitters depends on the activity of presynaptic VGCCs. Excessive glutamate activity, due to either excessive release or insufficient uptake from the synapse, leads to a condition called excitotoxicity. This pathological state is common among all neurodegenerative disorders, such as Alzheimer’s and Parkinson’s diseases. Under these conditions, glutamate adversely affects the trisynaptic circuitry, leading to synaptic destruction and loss of memory and learning performance. This study attempts to clarify the role of presynaptic VGCCs in memory performance and reveals that modulating the activity of presynaptic calcium channels in the trisynaptic pathway can regulate the excitotoxic state and consequently prevent the elimination of neurons and synaptic degradation. All of these can lead to an improvement in learning and memory function. In the current study, two calcium channel blockers—omega-agatoxin-Aa2a and omega-Lsp-IA—were extracted, purified, and identified from spiders (*Agelena orientalis* and *Hogna radiata*) and used to modulate N and P/Q VGCCs. The effect of omega-agatoxin-Aa2a and omega-Lsp-IA on glutamate-induced excitotoxicity in rats was evaluated using the Morris water maze task as a behavioral test. The local expression of synaptophysin (SYN) was visualized for synaptic quantification using an immunofluorescence assay. The electrophysiological amplitudes of the field excitatory postsynaptic potentials (fEPSPs) in the input-output and LTP curves of the mossy fiber and Schaffer collateral circuits were recorded. The results of our study demonstrated that N and P/Q VGCC modulation in the hippocampus trisynaptic circuit of rats with glutamate-induced excitotoxicity dysfunction could prevent the destructive consequences of excitotoxicity in synapses and improve memory function and performance.

## Introduction

The hippocampus is an area in the mammalian brain with a very intricate, extensive, and simultaneously precise function. The hippocampus has a variety of duties, including but not restricted to gathering massive amounts of information from internal and external body environments, as well as separating, classifying, encoding, storing, consolidating, and recalling them (Opitz, 2014). This crucial performance relies on neural circuits. The hippocampus consists of the dentate gyrus, cornu ammonis1 (CA_1_), CA_2_, and CA_3_, and the subiculum (Kjonigsen et al., 2011). Neural circuits of the hippocampus are categorized into direct and indirect types. The indirect pathway is called the trisynaptic pathway, because of the existence of three neurons in it, which transfer information from the entorhinal cortex to the CA_1_ area. The trisynaptic pathway includes three connection components. The entorhinal cortex neurons provide a perforant path to create excitatory synapses of the dentate gyrus granule cells. The granule cells proceed through the Mossy fiber pathway and make excitatory synapses with the pyramidal cells in the CA_3_ of the hippocampus. The CA_3_ cells excite the CA_1_ pyramidal cells through the Schaffer collateral pathway (Stepan et al., 2015; Chao et al., 2022). The facilitation of signal transduction in this pathway is significantly related to episodic memory (Hainmueller and Bartos, 2020; Salehifard et al., 2023). Spatial memory is a subcategory of episodic memory (Stepan et al., 2015; Hainmueller and Bartos, 2020). The episodic memory fluctuations and changes in experimental groups can be studied through spatial memory assessments, such as the Morris water maze test.

Glutamate is a primary excitatory neurotransmitter in the trisynaptic pathway and this neurotransmitter transmits information through this pathway (Bischofberger et al., 2006; Stepan et al., 2015). Therefore, in normal conditions, glutamate has a fundamental role in learning and memory via synapse firing, which in turn leads to synaptic plasticity (Dobrek and Thor, 2011). There are two classes of glutamate receptors. The first class of these receptors comprises ionotropic receptors, including α-amino-3-hydroxy-5-methyl-4-isoxazole propionic acid (AMPA), kainic acid, and N-methyl D-Aspartate receptors (NMDAR). The second class consists of metabotropic receptors, which couple with G-proteins (Dogra and Conn, 2021). Following the release from the presynaptic neuron into the synaptic cleft, the excitatory neurotransmitter binds to glutamate receptors. Balanced stimulation of glutamate receptors, particularly NMDARs, is indispensable for learning and memory formation through the long-term potentiation (LTP) process (Hayashi, 2022). LTP plays a crucial role in increasing dendrites and synaptic plasticity in the brain (Bin Ibrahim et al., 2022). Overstimulation of these receptors by agonists, such as NMDA, glutamate, aspartate, and kainic acid, can lead to destructive reactions in neuronal cells (Jarrard, 2002; Zhang et al., 2014; Hosseini-Sharifabad et al., 2021; Keimasi et al., 2022; Keimasi et al., 2023a). This hyper-stimulation may be due to the over-activity of the presynaptic voltage-gated calcium channels (VGCCs), which are involved in glutamate release (Schurr, 2004; Nimmrich and Gross, 2012; Sousa et al., 2013; Mochida, 2019).

Overstimulation of glutamate receptors allows excessive amounts of calcium into the neurons, which in turn leads to inflammation and neuronal damage (Arundine and Tymianski, 2003). On the other hand, calcium accumulation in mitochondria results in the production of numerous free radical species and triggers the apoptosis internal pathway (Szydlowska and Tymianski, 2010). These events lead to the induction of apoptosis in nerve cells, resulting in neuronal death, the elimination of neurotrophic factors, and synaptic plasticity (Verma et al., 2022; Salehifard et al., 2023). Many studies have indicated that the overactivation of glutamate receptors is essential for the development and progression of neurotoxicity and neurodegenerative disorders, known as excitotoxicity (Dong et al., 2009; Mehta et al., 2013; Lai et al., 2014). This phenomenon leads to neuronal dysfunction, synaptic degeneration, and the elimination of synaptic proteins, including synaptophysin (SYN) (Zhang et al., 2014; Ji et al., 2017). Intracellular calcium concentration control is an important issue during excitotoxicity as this balance can prevent the destructive effects of excitotoxicity on the neuronal cells as well as alleviate them.

According to several studies regarding neurotoxic models, the overstimulation of NMDA receptors is a consequence of NMDA administration in the rat hippocampus (Jarrard, 2002; Gilmour et al., 2012; Morland and Nordengen, 2022). This is similar to the excitotoxicity conditions in Alzheimer’s disease (AD) (Esposito et al., 2013; Ong et al., 2013). Dementia is described as a deterioration in cognitive function and is currently recognized as the seventh leading cause of death and one of the main causes of disability, particularly among the elderly (Organization, 2021). AD is the most common type of dementia. The intense scientific interest in AD represents the high prevalence of this progressive neurodegenerative disorder (Fahanik-Babaei et al., 2022). AD is identified by the progressive deprivation of memory and cognitive deficits (Kumar and Singh, 2015; Naseri et al., 2022). This disease imposes a wide range of direct and indirect costs on people and is highly prevalent across the world (Sarlaki et al., 2022). The financial burden of AD and related dementias (ADRDs) is anticipated to rise rapidly with the aging global population aging (Nandi et al., 2022). It is estimated that the global costs of dementia will escalate to US$ 1.7 trillion by 2030. If informal care costs are taken into account, this is projected to rise 60% more (US$ 1.7 trillion) (Organization, 2021). These all represent the importance of finding new ways to treat or prevent the progression of dementia, particularly AD, on economic and health grounds.

VGCCs are categorized into two major types: high voltage-activated (L, P/Q, N, and R) and low voltage-activated (T) VGCCs (Shafer and Meyer, 2004; Mochida, 2019). Presynaptic VGCCs (N and P/Q) are expressed in the nerve terminal and trigger docking, resulting in the merging of synaptic vesicles with presynaptic membranes through calcium entering the neuron cytoplasm. This mediates the release of neurotransmitter into the synaptic cleft (Atlas, 2001; Mochida, 2019). Consequently, presynaptic VGCC modulation can have significant effects on the excitotoxic state. Synaptophysin (SYN) is a synaptic vesicle protein that participates in synaptic transmission with a key role. This protein can be used for the quantification of synapses (Valtorta et al., 2004; Beiki et al., 2021). The rate of the effect of presynaptic VGCCs on synapses and episodic memory in the trisynaptic circuit can be evaluated by measuring local SYN protein expression.

The P/Q- and N-type VGCC modulators or blockers can bind to presynaptic VGCCs in various binding sites. For example, some drug agents bind to the alpha-2 delta subunit, whereas some bio-active small proteins such as omega-agatoxins, omega-lycotoxins, and omega-conotoxins seem to interact with the alpha-1 subunit (Nimmrich and Gross, 2012). In the present study, omega-agatoxin-Aa2a and omega-Lsp-IA were extracted and identified from the venom of two types of spiders (*Agelena orientalis* and *Hogna radiata*).

Venom is a complex biochemical compound that is produced and stored in living organisms, such as snakes, scorpions, lizards, bees, and spiders. For thousands of years, venom has been traditionally used to treat inflammation, joint pain, and arthritis in Chinese, Indian, and Egyptian medicine (Moradi et al., 2018). High solubility, low molecular weight, acceptable stability (due to the presence of disulfide bonds), synthesizability, and selectivity in binding to target receptors are some of the advantages of venom peptides. Hence, venom-modulatory proteins and peptides are highly selective for specific channels, and they can also target ion channels and receptor-coupled G proteins (Lewis and Garcia, 2003).

The UniProt database has shown that the venoms of the Lycosidae and Agelenidae spider families contain high levels of Lycotoxin (Liu et al., 2009) and Agatoxin proteins (Consortium, 2019), which are related to L-, P/Q-, and N-type VGCC modulators and blockers (Nimmrich and Gross, 2012; Sousa et al., 2013). Therefore, omega-Lsp-IA and omega-agatoxin-Aa2a can be considered as P/Q-type and N-type VGCC modulators, respectively (Consortium, 2019; Keimasi et al., 2022; Keimasi et al., 2023a).

This study aimed to assess the effects of neurotransmitter release on episodic memory under normal and excitotoxic conditions in the hippocampal trisynaptic circuit. To achieve this goal, a variety of behavioral, molecular, and electrophysiological methods were used. The crude venoms for this study were extracted from two types of spiders (*Agelena orientalis* and *Hogna radiata*). Omega-Lsp-IA and omega-agatoxin-Aa2a were identified and purified from the extracted crude venoms. These bio-active proteins were used together as P/Q- and N-type VGCC modulators in the character of co-treatment. A behavioral test (Morris water maze) was used to evaluate long-term and spatial memories. The electrophysiological amplitude of field excitatory postsynaptic potentials (fEPSPs) in the input-output and LTP curves was also recorded in the mossy fiber and Schaffer collateral pathways. Subsequently, we measured the amount of SYN for synaptic quantification using an immunofluorescence technique.

## Materials and methods

### Chemicals, reagents, and buffers

All chemicals, except those mentioned in the text, were purchased from Sigma-Aldrich (Darmstadt, Germany).

### Spider collection, identification, and venom extraction

For this study, live specimens were collected from Iran (Zamani et al., 2021). Suitable conditions in terms of humidity and temperature were provided for the specimens. They were fed with crickets and mealworms. Taxonomic keys (particularly by epigyne of female spiders) were used to identify the specimens (Nentwig et al., 2017; Keimasi et al., 2023a; Keimasi et al., 2023b).

Female spiders (*Agelena orientalis* and *Hogna radiata*) were separated for venom extraction. Specimens were anesthetized with CO_2_ in a small chamber, and the opisthosoma and carapace were removed under a stereomicroscope. Venom glands collected in phosphate-buffered saline (PBS) at 4°C were prepared in the laboratory, using 137 mM NaCl, 3 mM KCl, 10 mM Na_2_PO_4_, and 2 mM KH_2_PO_4_ (pH 7.4). The collected glands were gently crushed for 30 min with a glass stirrer. Subsequently, the pieces of the venom glands were removed from the solution by centrifugation at 13,000 rpm for 30 min at 4°C. The supernatant was lyophilized and stored at −70°C. The concentration of the protein was measured by Bradford assay using bovine serum albumin (BSA) as the standard protein. The tissue samples and vouchers were stored in the Zoological Museum, University of Isfahan (ZMUI).

### Protein purification using gel-filtration chromatography

To purify protein with gel-filtration chromatography, initially, 10 mg of each lyophilized crude venom (*Agelena orientalis* and *Hogna radiata*) was resuspended in 1.5 mL PBS buffer. The samples were incubated for 2 h at 4°C following the addition of RNase (0.14 mg/mL) and DNase (0.14 mg/mL) enzymes. Then, the plain solution was injected into a GE Healthcare HiLoad 16/600 Superdex^®^ 75 pg prep grade gel-filtration column and ran over it using a fast protein liquid chromatography (FPLC) system (Sykam, Germany). PBS Buffer was used for washing the column. A flow rate of 0.7 mL/min and an injection volume of 1,200 μL were used. Absorbance at 280 nm was obtained for the fractions, which were then accumulated in a 0.75 mL fraction. The selected fractions, marked on the graph, were accumulated and injected into a capillary electrophoresis instrument (Keimasi et al., 2022; Keimasi et al., 2023a).

### Protein purification and separation with capillary electrophoresis

The Agilent 7100 system with a UV-Vis detector, a 40 cm, 50 μm uncoated silica column, and a detector distance of 8.5 cm from the outlet was used for the capillary electrophoresis test. For the sample, and the running buffer, PBS buffer (pH 4.7) was used. The sample was injected at a pressure of 100 mBar for 5 s at a capillary temperature of 25°C. Electrophoresis was performed at 25 kV normal polarity for 5 and 15 min for *Agelena orientalis* and *Hogna radiata*, respectively. Following accumulation of the labeled peaks, a Bradford assay was used to identify the protein concentration. To ensure purity, the selected peak was reinjected into the device. The resulting peaks were injected into an HPLC-ESI-MS instrument thereafter (Keimasi et al., 2022; Keimasi et al., 2023a).

### Protein identification with mass spectrometry (HPLC-ESI-MS)

A Waters Alliance 2695 HPLC-Micromass Quattro micro API Mass Spectrometer was used to perform high-performance liquid chromatography/electrospray ionization tandem mass spectrometry (HPLC-ESI-MS) analysis. An Atlantis T3-C18 column (3 µ, 2.1 × 100 mm) was used for liquid chromatography at 35°C. Formic acid (0.1%) in acetonitrile (A) and 0.1% formic acid in H_2_O were used as mobile phases. With a 0.2 min held, the gradient profile was 5% which increased linearly to 90% in 10 min. Thereafter, it was held for 5 min and dropped to 5% over 3 min. At the end, it was held for 4 min. An injection volume of 5 μL and a 0.2 mL/min flow rate were used for the analysis. A positive mode and a 0.3 kV capillary voltage were used for the mass spectrometry. A gas flow of 200 L/h, 300°C dissolving, and 120°C source were used in the study. The outcome is shown for the purified bio-active small protein peaks (Keimasi et al., 2022; Keimasi et al., 2023a).

### Animals and experimental design

A total of 36 adult male Wistar rats with a weight range of 230–250 g were selected from the animal nest of the Faculty of Biological Sciences and Technology, University of Isfahan. Standard cages were used to retain them at 22°C and 60% humidity under a 12-h light-dark cycle with free access to enough food and water. All the animal procedures were approved by The University of Isfahan’s animal ethics committee under the research code number 16192.

This was a study on male Wistar rats, separated accidentally into three groups of twelve rats. After fixing the head with a stereotaxic instrument, to prepare for hippocampus injection, a small area on the skull of each rat was shaved. As a vehicle for omega-Lsp-IA, omega-agatoxin-Aa2a, and N- Methyl d-Aspartate (NMDA), PBS was used. The rats were assigned to the subsequent groups: the control group with a hippocampus injection of 2 µL PBS followed by 2 µL PBS intrahippocampal injection after 30 min; the NMDA-treated group with a 2 µL injection of PBS followed by a single intrahippocampal injection of NMDA (10 μg, 5 μg/μL) after 30 min; the omega-Lsp-IA and omega-agatoxin-Aa2a co-treated group, which received a single dose of NMDA (10 μg, 5 μg/μL) followed by an injection of 1 µL of omega-Lsp-IA (2 μg, 2 μg/μL) and 1 µL of omega-agatoxin-Aa2a (2 μg, 2 μg/μL) as co-treatment in the hippocampus, 30 min later.

### Surgery and microinjection procedure

All the rats were deeply anesthetized with an intraperitoneal phenobarbital (40 mg/kg) injection [purchased from Martindale Pharma Company (Buckinghamshire, England)], 1 week ahead of the behavioral tests. For the operation, the examined rats were enfolded with towels, while their eyes were shielded with Vaseline. Then, the Paxinos and Watson rat brain atlas was used to identify their hippocampus area (Paxinos and Watson, 2006). The injection was performed bilaterally on the hippocampus (AP: −3.3 mm from bregma; ML: ±2.5 mm from midline; DV: −3.2 mm from the skull surface) using a stereotaxic apparatus (Stoelting Co., United States).

A 5 µL Hamilton syringe with an injection needle (21 gauge), connected using a polyethylene tube, was used to inject the agents into hippocampi, bilaterally. This injection was carried out within 6 min. To avoid any probable fluid backflow, the needle was gently removed 2 min after the injection.

### Behavioral study

After 1 week from the stereotaxic surgery (Morris water maze tasks), the behavioral studies were implemented. These tests assessed spatial and long-term memory and learning. To get adjusted to the experimental conditions and stress reduction, the animals were acclimatized in the laboratory for 2 days before the behavioral assessment.

### Morris water maze task

The Morris water maze test was performed to evaluate spatial memory and learning. In this test, the rats used their spatial memory to find the hidden platform in the target zone, according to the signs and symbols on the water maze room walls. This test consists of three steps: habituation, training, and test (previously described by Hosseini-Sharifabad et al., 2021). The time spent, distance moved, frequency of entry into the target quadrant, and swimming paths of the rats during the probe trial were documented to evaluate spatial memory and learning. One-way and two-way ANOVA tests were performed to analyze the collected data.

### Electrophysiological studies

The interaction between P/Q- and N-type VGCCs and memory in excitotoxicity conditions were studied by fEPSP. Afterward, the rats were sacrificed in the CO_2_ chamber, and their brains were removed using a guillotine. Then, the hippocampus was detached using surgical instruments and frozen in liquid nitrogen and finally stored in a −70°C freezer, except for the brains that were removed for the histology section.

The mossy fiber circuit is part of the trisynaptic pathway. In this circuit, information from the dentate gyrus reaches the CA_3_. This synaptic firing has a vital role in memory formation. The input-output LTP was performed as described in a previous study (Radahmadi et al., 2023). Urethane in normal saline (1.5 g/kg; intraperitoneal injection) was used to anesthetize the rats (six per each group). The hippocampal CA_3_ surgery and LTP recording procedures were performed as described previously by Keimasi et al. (2022) (Keimasi et al., 2022).

The Schaffer collateral circuit is the last piece of the puzzle in the trisynaptic pathway. During this circuit, information is transferred from CA_3_ to CA_1._ This transfer is necessary to complete the information for memory formation. LTP of the CA_1_ region was performed considering the Radahmadi et al. (2023) study, with six rats in each group, and the baseline recordings were taken 60 min after high-frequency stimulation (HFS) (Radahmadi et al., 2023). A one-way repeated measures ANOVA test was used to analyze the collected data.

### Immunofluorescence staining

Saline was used to wash the rat brains. Thereafter the samples (the hippocampi of the rats) were fixed in 10% formaldehyde solution. Subsequently, a microtome instrument was used to cut the samples into 2-µm segments. Enzymatic antigen retrieval was executed for 20 min. The samples were blocked using a blocking agent [10% normal goat serum (Sigma, G9023) and 0. 3% Triton X-100 in PBS] for 30 min at 37°C to lessen non-specific antibody binding. Following the washing of the tissue sections, they were incubated with mouse anti-synaptophysin monoclonal antibody (Abcam, ab8049) as the primary antibody, diluted 1:1000 in PBS at 4°C overnight. As the secondary antibody, FITC-conjugated anti-mouse IgG (Sigma, F9137) was used at a dilution of 1:1000 for 2 h at room temperature. The nuclei were counterstained using 4′, 6-diamidino-2-phenylindole (DAPI, Sigma, D9542). An AX70 Olympus fluorescence microscope was used to visualize the slides (Keimasi et al., 2023a). All cells in the merge section were counted, and then the positive SYN cells were counted. Next, the percentage of positive SYN cells from the total cells was calculated. Finally, one-way and two-way ANOVA tests were performed to analyze the collected data.

### Data analysis

GraphPad Prism statistics software (version 8.4.3) was used to perform the statistical analyses. The D'Agostino-Pearson omnibus test was used to check the normality of the statistical data. One-way, one-way repeated measures and two-way ANOVA were performed to analyze the collected data. Tukey’s test was performed for multiple comparisons. The resulting data are shown as mean ± standard error.

## Results

### Spider collection, identification, and venom extraction

Following a collection of 65 spiders from nature, they were separated according to their species and gender. For the next phase of the research, 15 female specimens of *Hogna radiata* and 30 female specimens of *Agelena orientalis* were separated.

From each group of specimens, 150 mg of the lyophilized crude venom was obtained. Next, PBS buffer was used to dissolve 10 mg of crude venom from each species. The protein concentrations of the lyophilized crude venoms were evaluated using the Bradford method and found to be 6.9 mg/mL and 6.7 mg/mL for *Agelena orientalis* and *Hogna radiata*, respectively.

### Protein purification with gel-filtration chromatography

**FIGURE 1:**
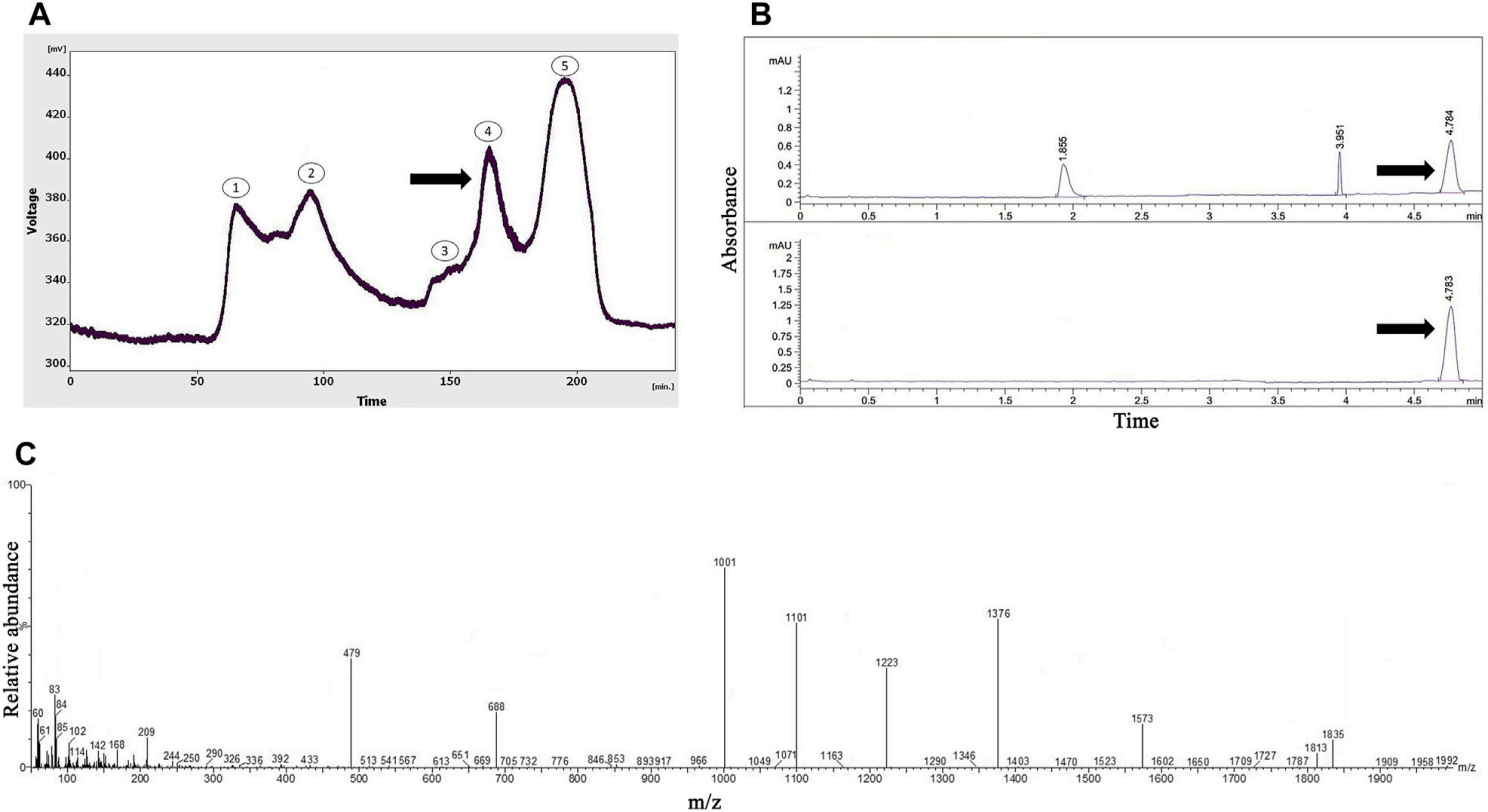
The purification and identification of omega-agatoxin IIA from the *Agelena orientalis* crude venom. **(A)** Gel-filtration chromatography of the crude venom performed on a GE Healthcare HiLoad 16/600 Superdex^®^ 75 pg prep grade column in 1 M PBS (pH 7.4), with a flow rate of 0.7 mL/min. The fourth fraction is shown by a black arrow. **(B)** Capillary electrophoresis of the fifth fraction was performed on a 50 μm uncoated silica column in 1 M PBS (pH 4.7) for 5 min. The omega-agatoxin IIA fraction is shown by black arrows. **(C)** HPLC-ESI-MS of the omega-agatoxin IIA fraction was performed on an Atlantis T3-C18 column. The spectrum results are shown in part **(C)**

As presented in Figure 1A, five peaks were revealed following the gel-filtration chromatography of the *Agelena orientalis* venom; among them, the fourth fraction of *Agelena orientalis* venom was collected from 155 to 180 min. From 17.5 mL of solution yielded from the device, a 1.62 mg lyophilized fraction was obtained with a protein concentration of 1.01 mg/mL.

**FIGURE 2:**
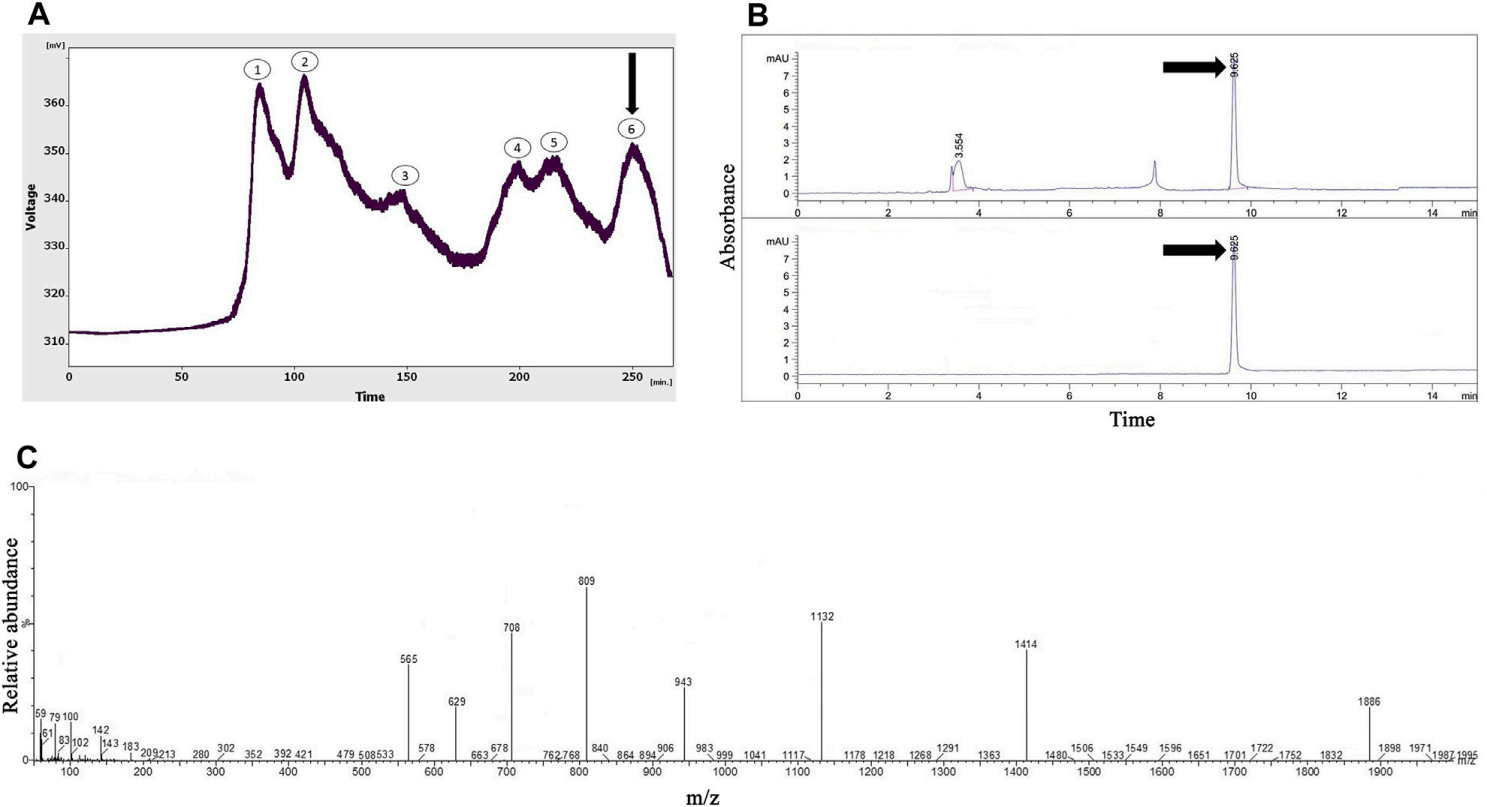
The purification and identification of omega-Lsp-IA from the *Hogna radiata* crude venom. **(A)** Gel-filtration chromatography of the crude venom performed on a GE Healthcare HiLoad 16/600 Superdex^®^ 75 pg prep grade column in 1 M PBS (pH 7.4), with a flow rate 0.7 mL/min. The sixth fraction is shown by a black arrow. **(B)** Capillary electrophoresis of the sixth fraction was performed on a 50 μm uncoated silica column in 1 M PBS (pH 4.7) for 10 min. The omega-Lsp-IA fraction is shown by black arrows. **(C)** HPLC-ESI-MS of the omega-Lsp-IA fraction was performed on an Atlantis T3-C18 column. The spectrum results are shown in part **(C)**

As shown in Figure 2A, six peaks were obtained from the gel-filtration chromatography of the *Hogna radiata* venom; among them, the sixth fraction was collected from 240 to 260 min. From the 14 mL solution taken from the instrument, a 1.47 mg lyophilized fifth fraction was obtained with a concentration of 1.08 mg/mL (determined using the Bradford method).

### Protein purification with capillary electrophoresis (CE)

Figure 1B displays the pattern obtained with the fourth fraction of *Agelena orientalis* venom.

Three peaks were observed at 1.8, 3.9, and 4.7 min. The graph shows narrow peaks. The first peak was collected and reinjected to confirm the purity of this small bioactive protein and then injected into HPLC-ESI-MS for mass determination.

Figure 2B shows the pattern obtained from the sixth fraction of *Hogna radiata* venom, using the same technique, in which three peaks were observed at 3.5, 7.8, and 9.6 min. The third peak was injected into HPLC-ESI-MS for mass determination.

### Protein identification with mass spectrometry (HPLC-ESI-MS)

The molecular mass of omega-agatoxin-Aa2a is 10,982 Da, according to a previous study (Sousa et al., 2013). The spectrum results indicated the presence of omega-agatoxin-Aa2a in the chosen peak (Figure 1C). The mass-to-charge ratio of this bio-active small protein was quite consistent with omega-agatoxin-Aa2a. The charge-to-mass ratio of omega-agatoxin-Aa2a was as follows: (M + Na + 5H)^6+^ = 1,835, (M + Na + 3H)^4+^ = 769.5, and (M + Na + 4H)^5+^ = 616.

In agreement with a previous study (Pluzhnikov et al., 2007), the molecular mass of omega-Lsp-IA was determined to be 5,631.5 Da. The results of the mass spectrum showed the presence of omega-Lsp-IA (Figure 2C). The mass-to-charge ratio of this protein was quite congruent with omega-Lsp-IA. The charge-to-mass ratio of omega-Lsp-IA was as follows: **(**M + Na+2H)^3+^ = 1,886.1, **(**M + Na + 3H)^4+^ = 1,414.37, and (M + Na + 5H)^6+^ = 943.25.

### Morris water maze

**FIGURE 3:**
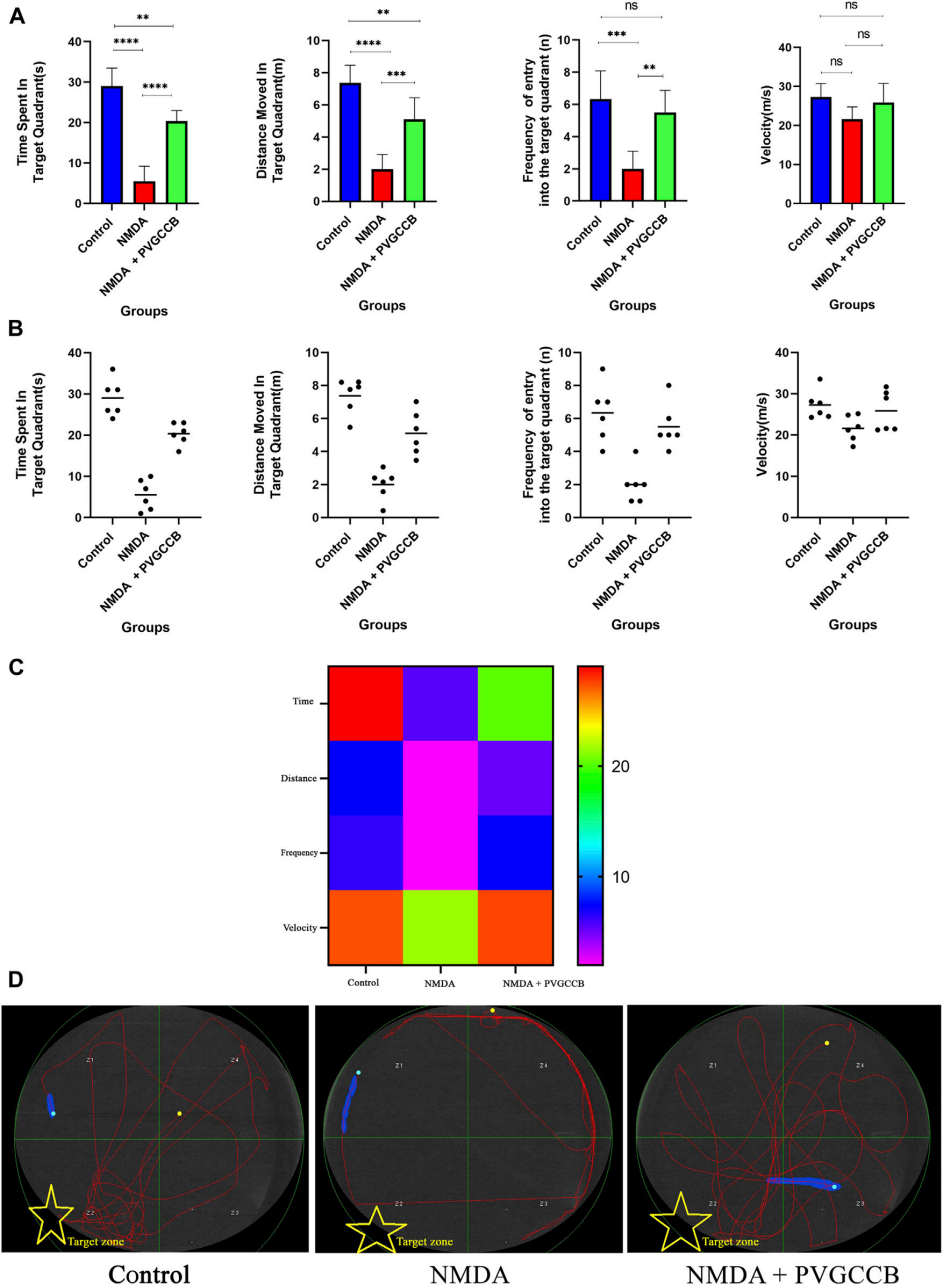
The Morris water maze task. **(A)** The effect of treatment with omega-Lsp-IA and omega-agatoxin IIA on time spent, distance moved, frequency of entry into the target quadrant, and the velocity of rat swim. The data are shown as mean ± SEM of six rats per group (one-way ANOVA). *p < 0.05, **p < 0.01, ***p < 0.001, ****p < 0.0001. **(B)** Scatter plot of part **(A)**. **(C)** Heat map graph for Morris water maze technique indicators in the target zone and the velocity (two-way ANOVA). **(D)** The swim path traces from the test day. The target zone is indicated with yellow stars. PVGCCB, presynaptic voltage-gated calcium channel blockers.

As shown in Figures 3A, B, injection of NMDA in the hippocampus markedly decreased the time spent (*p* < 0.0001), distance moved (*p* < 0.0001), and frequency (*p* < 0.001) of entry into the target quadrant of the maze in the NMDA-treated group, when compared with the control group. The NMDA-treated group with a single dose of presynaptic VGCC blocker noticeably spent more time (*p* < 0.0001), moved a greater distance (*p* < 0.001), and entered the target quadrant of the maze more frequently (*p* < 0.01) compared with the NMDA-treated group. A significant difference in the time spent (*p* < 0.01) and distance moved (*p* < 0.01) in the target quadrant of the maze was observed between the control and NMDA-treated + presynaptic VGCC blocker groups (one-way ANOVA).

Figure 3C shows the heat map graph of the Morris water maze technique indicators in the target zone and the velocity. This provides a better presentation of the significant difference between the groups compared (two-way ANOVA).

The path of the control rats showed a regular round movement, whereas the swimming path of the NMDA-treated rats in the target area was irregular with no particular pattern. The swimming path of NMDA-treated + presynaptic VGCC blocker rats was similar to the control group (Figure 3D).

### Mossy fiber circuit LTP

**FIGURE 4:**
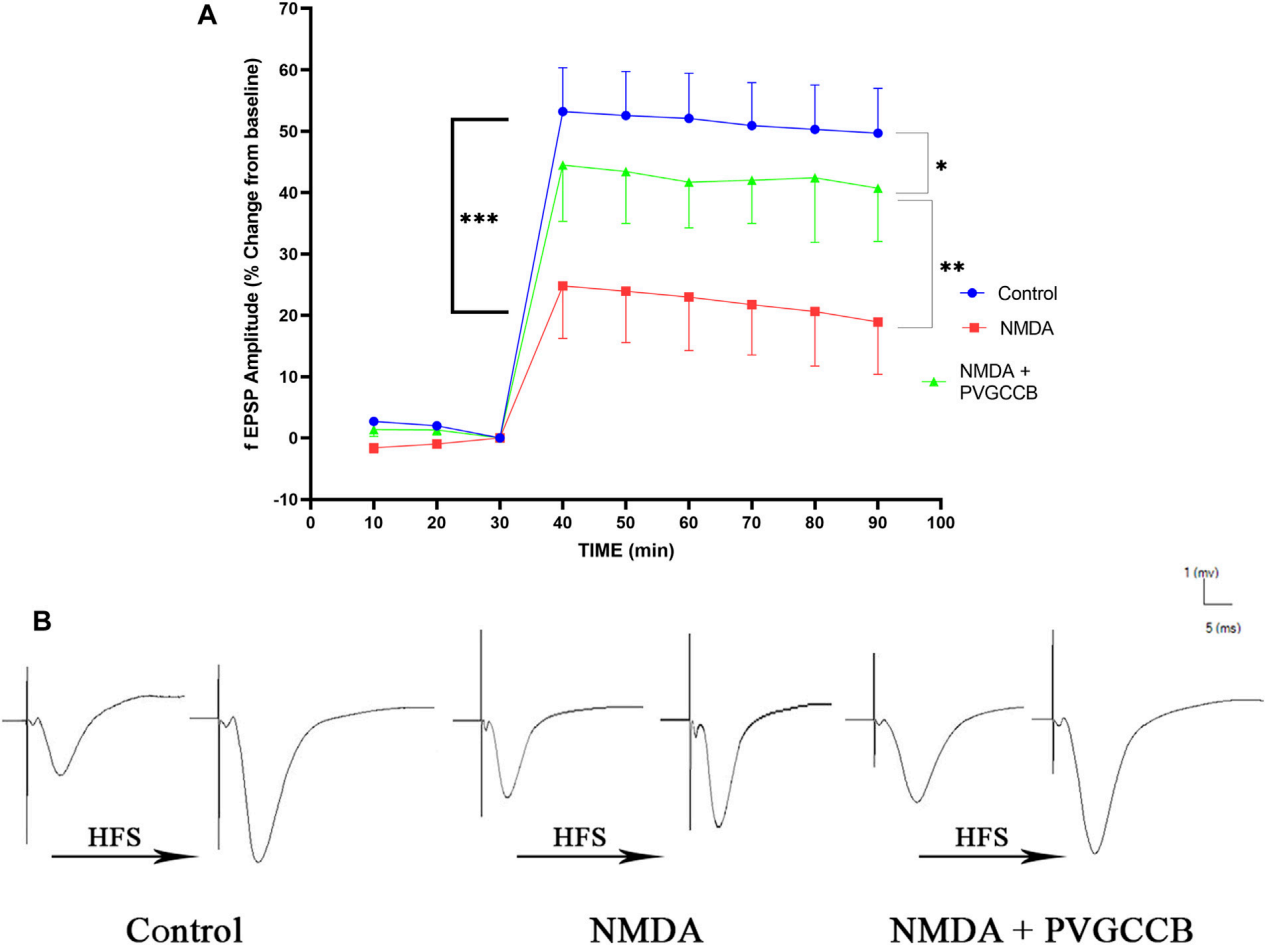
Mossy fiber circuit LTP. **(A)** Long-term potentiation (LTP) curves of the field excitatory postsynaptic potential (fEPSP) amplitude in the hippocampal CA_3_ for all groups (*n* = 6). The data are mean ± SEM of six rats per group (one-way ANOVA repeated measures). *p < 0.05, **p < 0.01, ***p < 0.001, ****p < 0.0001, and ^####^ *p* < 0.0001. **(B)** Sample traces of typical recorded fEPSPs in the hippocampal CA_3_ neurons before and after high-frequency stimulation (HFS) induction for long-term potentiation (LTP) in experimental groups. PVGCCB, presynaptic voltage-gated calcium channel blockers.

Taking into account Figure 4A, NMDA injection considerably reduced the field excitatory postsynaptic potentials (fEPSP) amplitude in the NMDA-treated group after LTP induction in the hippocampus CA_3_, in comparison with the control group (*p* < 0.001). Following NMDA injection in the NMDA-treated + presynaptic VGCC blocker group, the administration of presynaptic VGCC blockers increased the fEPSP amplitude following LTP induction when compared with the NMDA-treated group (*p <* 0.01). The fEPSP amplitude in the NMDA-treated + presynaptic VGCC blocker group showed a significant difference compared with the control group (*p* < 0.05).

Figure 4B shows the traces of the recorded fEPSPs in the CA_3_ neurons, before and after LTP induction with the use of a high-frequency stimulation (HFS) technique in all the compared groups.

### Schaffer collateral circuit LTP

**FIGURE 5:**
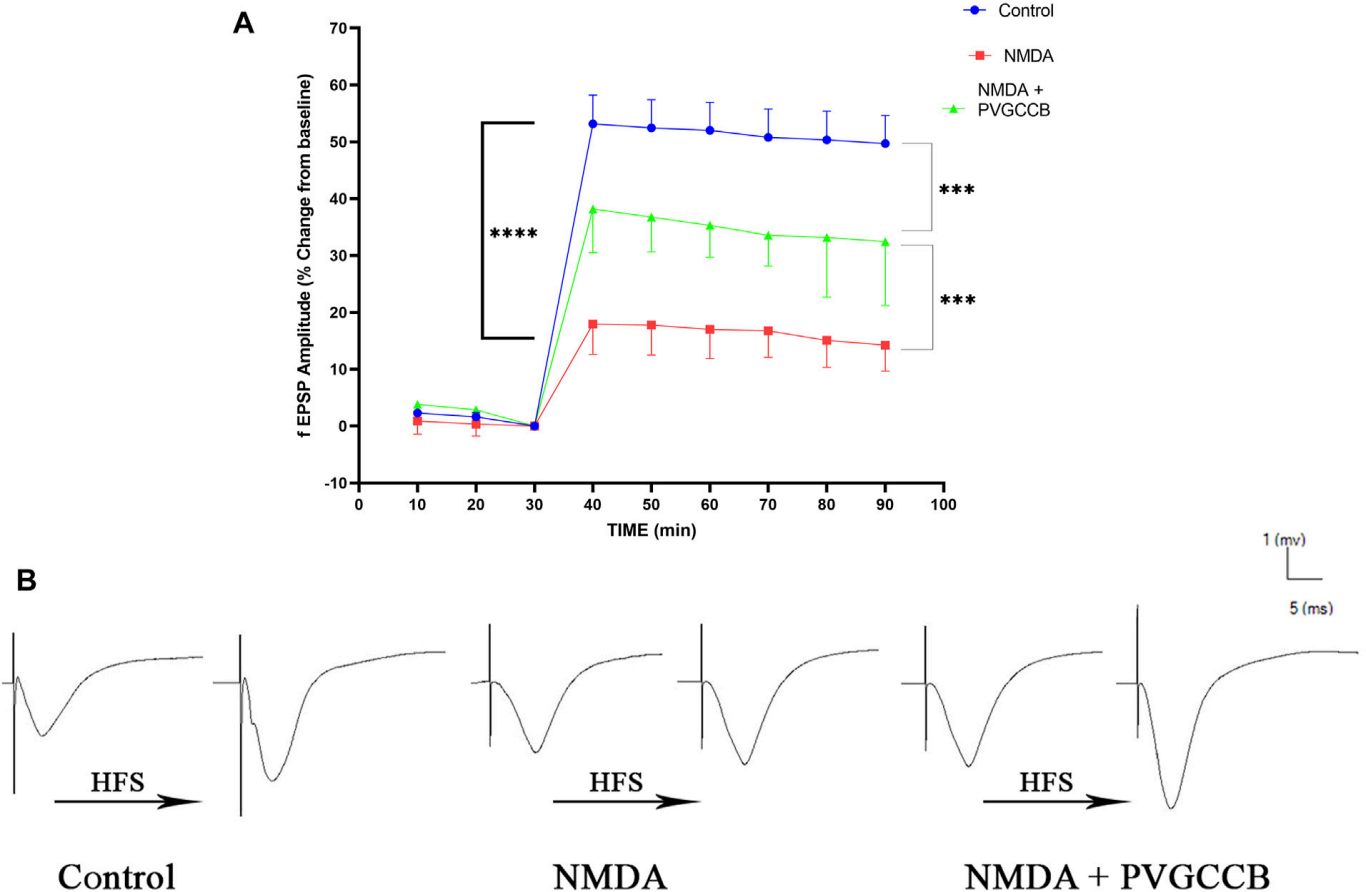
Schaffer collateral circuit long-term potentiation (LTP). **(A)** LTP curves of the field excitatory postsynaptic potential (fEPSP) amplitude in the hippocampal CA_1_ for all groups (n = 6). The data are mean ± SEM of six rats per group (one-way ANOVA repeated measures). *p < 0.05, **p < 0.01, ***p < 0.001, ****p < 0.0001), and ^####^ *p* < 0.0001. **(B)** Sample traces of typical recorded fEPSPs in the hippocampal CA_1_ neurons before and after high-frequency stimulation (HFS) induction for the LTP in experimental groups. PVGCCB, presynaptic voltage-gated calcium channel blockers.

As shown in Figure 5A, a single NMDA administration reduced the fEPSP amplitude in the NMDA-treated group following LTP induction in the CA_1_ when compared with the control group (*p* < 0.0001). The intra-hippocampal injection of omega-Lsp-IA and omega-agatoxin IIA as co-treatment following getting NMDA, amplified the fEPSP amplitude significantly following LTP induction when compared with the NMDA-treated group (*p* < *0.001*). The fEPSP amplitude in the NMDA-treated + presynaptic VGCC blocker group showed a significant difference in comparison with the control group (*p* < 0.001).

Figure 5B shows the traces of the recorded fEPSPs in the CA_1_ neurons, before and after LTP induction with the use of the HFS method among all the compared groups.

### Localized SYN in two circuits

**FIGURE 6:**
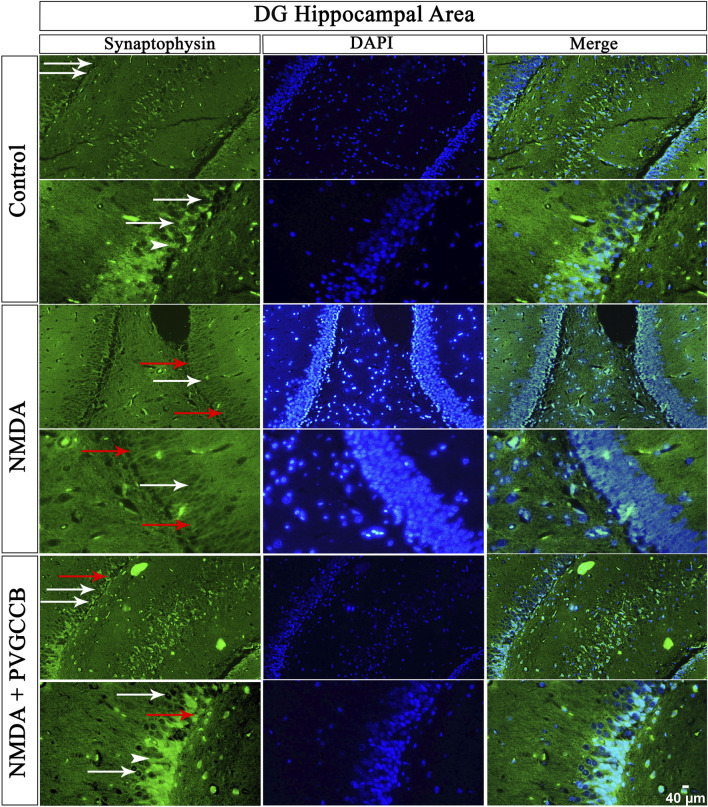
Immunofluorescence staining with an antibody against synaptophysin in the trisynaptic circuit. The localized synaptophysin is presented in the DG area of the rat hippocampus. DAPI was applied to counterstain the nuclei. Synaptophysin was used to show the synaptic plasticity (white arrowheads). The healthy (white arrows) and dead (red arrows) pyramidal neurons are indicated in the central column of each area. PVGCCB, presynaptic voltage-gated calcium channel blockers.

**FIGURE 7:**
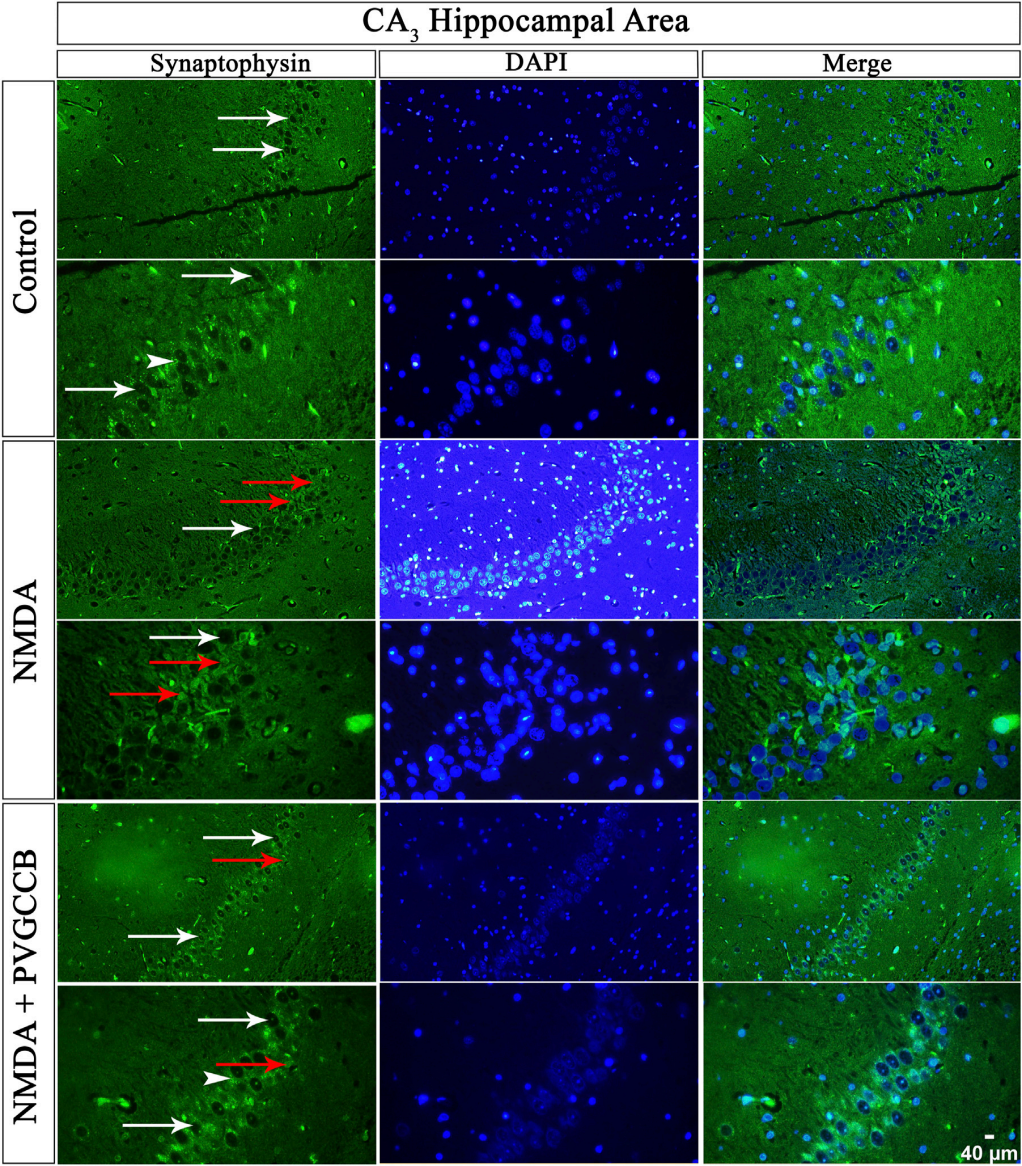
Immunofluorescence staining with an antibody against synaptophysin in the trisynaptic circuit. The localized synaptophysin is presented in the CA_3_ area of rat hippocampus. DAPI was applied to counterstain the nuclei. Synaptophysin was used to show the synaptic plasticity (white arrowheads). The healthy (white arrows) and dead (red arrows) pyramidal neurons are indicated in the central column of each area. PVGCCB, presynaptic voltage-gated calcium channel blockers.

**FIGURE 8:**
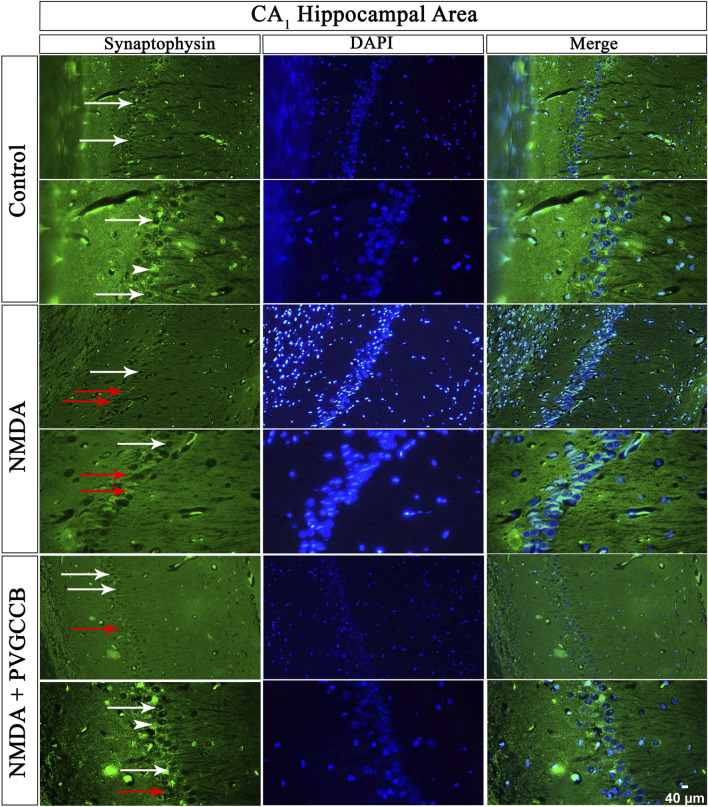
Immunofluorescence staining with an antibody against synaptophysin in the trisynaptic circuit. The localized synaptophysin is presented in the CA_1_ area of rat hippocampus. DAPI was applied to counterstain the nuclei. Synaptophysin was used to show the synaptic plasticity (white arrowheads). The healthy (white arrows) and dead (red arrows) pyramidal neurons are indicated in the central column of each area. PVGCCB, presynaptic voltage-gated calcium channel blockers.

**FIGURE 9:**
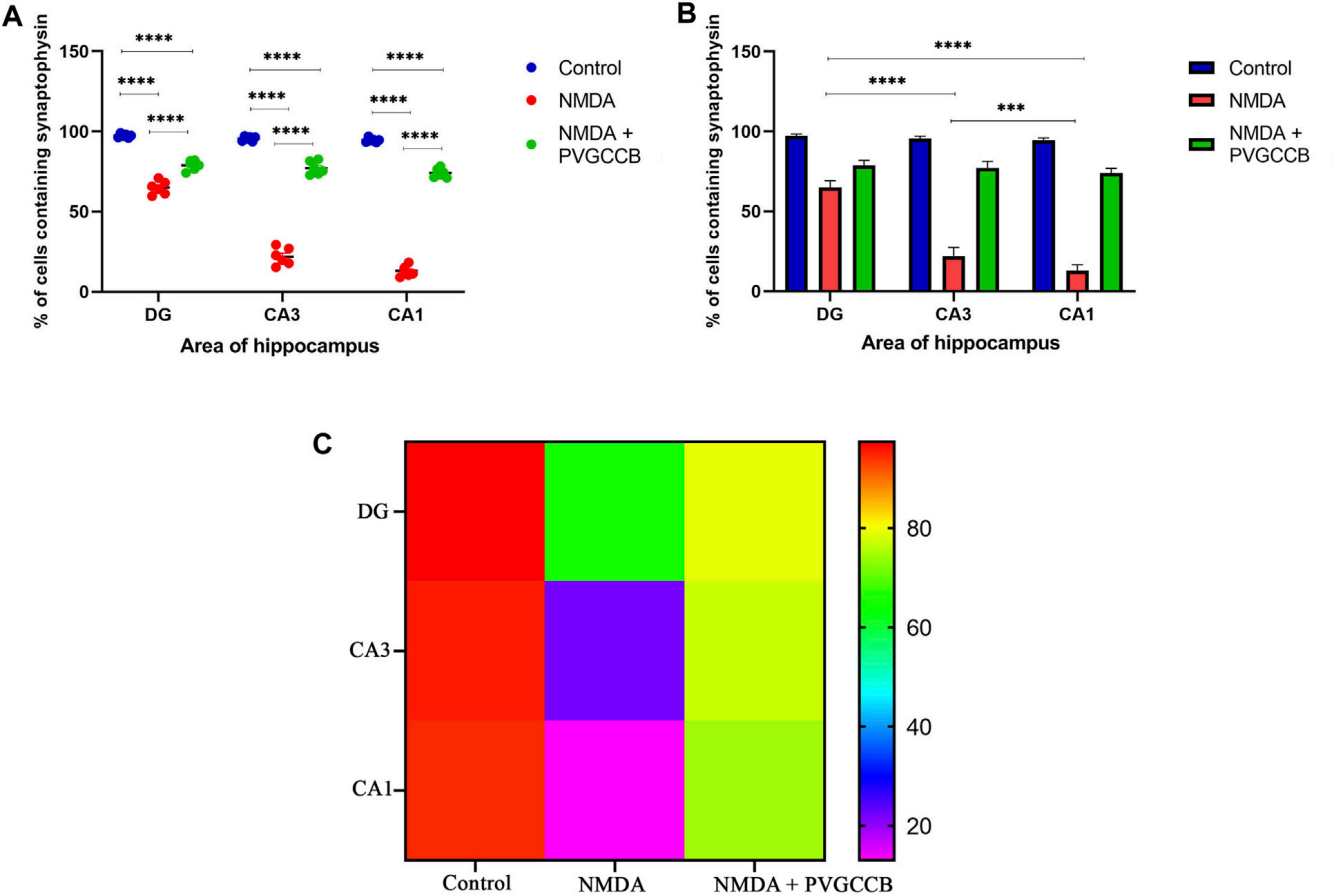
Immunofluorescence staining with an antibody against synaptophysin in the trisynaptic circuit. **(A,B)** The localized synaptophysin is shown in the DG, CA3, and CA1 areas of rat hippocampus for the experimental groups (%). The data are mean ± SEM of three rats (each rat, two sections) per group (two-way ANOVA). *p < 0.05, **p < 0.01, ***p < 0.001, ****p < 0.0001. **(C)** Heat map graph for the localized synaptophysin is shown in the DG, CA3, and CA1 areas of rat hippocampus for the experimental groups (two-way ANOVA). PVGCCB, presynaptic voltage-gated calcium channel blockers.

Our data showed that an NMDA single injection significantly reduced the expression of SYN protein in DG (*p* < 0.0001), CA_3_ (*p* < 0.0001), and CA_1_ (*p* < 0.0001) areas for the control group. The single administration of presynaptic VGCC blockers after injection of NMDA noticeably increased the expression of SYN in DG (*p* < 0.0001), CA_3_ (*p* < 0.0001), and CA_1_ (*p* < 0.0001) in comparison with the NMDA-treated group. The NMDA-treated + presynaptic VGCC blocker group showed significant differences in DG (*p* < 0.01), CA_3_ (*p* < 0.0001), and CA_1_ (*p* < 0.0001) compared with the control group (Figures 6–9, parts A, and B).

As shown in Figure 9B, there were significant differences between the NMDA-treated groups of different areas of the hippocampus. The expression of SYN protein in the DG area showed a significant difference with CA_3_ (*p* < 0.0001) and CA_1_ (*p* < 0.0001) areas. In addition, there was a significant difference between the CA_**3**_ and CA_**1**_ areas (*p* < 0.001). Figure 9C shows the heat map graph to provide a better understanding of the significant difference in the expression of SYN protein in hippocampus areas between the compared groups.

## Discussion

The current study investigated the role of presynaptic VGCC blockers (omega-Lsp-IA and omega-agatoxin-Aa2a) in explicit memory performance using the NMDA-induced excitotoxicity rat model. The crude venoms for this study were extracted from the spiders *Agelena orientalis and Hogna radiata*. Omega-Lsp-IA and omega-agatoxin-Aa2a were purified and identified from the extracted venoms. These bio-active proteins were used as co-treatment with P/Q- and N-types of VGCC modulators or blockers. The Morris water maze task was used in the current study. The electrophysiological amplitudes of field excitatory postsynaptic potentials (fEPSPs) were recorded before and after LTP induction in the mossy fiber, and Schaffer collateral pathways. Subsequently, the amount of localized SYN for synaptic quantification was measured using an immunofluorescence assay.

The concentration and amount of the obtained venom proteins were at acceptable levels. The protein concentration of these venoms were in line with some previous studies (Liu et al., 2009; Langenegger et al., 2019). *Hogna radiata* species are bigger and have larger venom glands than the *Agelena orientalis* species. Accordingly, the amount of venom obtained from *Hogna radiata* was much higher than that from *Agelena orientalis*.

The principle of separation was according to the connection of the analyte to the stationary and mobile phases. In the FPLC method, proteins and peptides were separated based on their molecular weights. Small peptides strongly interacted with the stationary phase, and larger peptides and proteins left the column sooner in the normal condition. The gel chromatography of *Agelena orientalis* venom provided several peaks. Considering the column type used, the fifth fraction proteins had the lowest mass. As a result, the fourth fraction was selected for the remainder of the study and injected into the CE device. The third peak was collected and reinjected to verify the purity of this small bioactive protein. This action confirmed not only the purity of this peak but also the accuracy of the collected peaks. The value of this peak was considerably greater than the other peaks. Figure 1 represents the purification and identification of omega-agatoxin-Aa2a from the *Agelena orientalis* crude venom. This protein was used as a selective antagonist for N-type VGCCs (Keimasi et al., 2023a). Silmara R Sousa et al. (2013) have pointed out the same function for omega-agatoxin-Aa2a in their review (Sousa et al., 2013).

The *Hogna radiata* gel-filtration pattern represented six separated peaks. The proteins in the last fraction had the lowest mass among all the fractions. Therefore, this fraction was selected for the next phase of the study. The general shape of the graphs of the two species indicated that the proteins of *Agelena orientalis* are smaller than those of *Hogna radiata*. Spiders of the *Agelenidae* family express large amounts of various agatoxin-type proteins, whereas the spiders in the Lycosidae family express proteins called lycotoxins in their venom. According to the *Arachnoserver* spider venom database, lycotoxins have a higher mass than agatoxins. Our findings from gel-chromatography were in accordance with the available data (Herzig et al., 2010).

Our findings confirmed the purification of a sufficient amount of omega-Lsp-IA and the accuracy of its detection from *Hogna radiata* venom. The omega-Lsp-IA protein is a modulator for P/Q-type VGCCs, based on the study by Pluzhnikov et al. (2007).

According to Figure 3, the data obtained from the Morris water maze test was supported by those of previous studies, which have demonstrated the glutamate-induced excitotoxicity effect on spatial memory defects and cognitive impairment (Hynd et al., 2004; Binvignat and Olloquequi, 2020; Liu et al., 2020).

As mentioned in the Introduction, spatial memory is a subset of episodic memory that can be well-tested with the Morris water maze task (Amanzadeh Jajin et al., 2021). In this test, the rats use clues on the wall when searching for the underwater platform. Additionally, they look around when they find the platform. This process is memorized by the hippocampus. The performance of all parts of the hippocampus is essential for completing this test.The NMDA-treated rats failed to complete this test, showing their cognitive impairment. This defect is caused by glutamate-induced excitotoxicity. This deficiency was eliminated mainly when presynaptic VGCC blockers were used. This improvement relies on the regulation of neurotransmitter release through presynaptic VGCC modulation (Hosseini-Sharifabad et al., 2021). The role of these presynaptic VGCCs becomes more prominent in such conditions. Additionally, this function indicates the importance of the amount of neurotransmitters released at the synapse. If the amount of glutamate released at the synapse is excessive, the effect of this neurotransmitter would be destructive (Zott and Konnerth, 2023). This is while the right amount of glutamate records external information, stores it, and memorizes it (Collingridge and Abraham, 2022). This dual role adds to the importance of glutamate release, following the presynaptic VGCC activity. The neurons in the trisynaptic pathway of the hippocampus can discriminate between high and normal amounts of glutamate and react differently accordingly. In normal conditions, these neurons have a firing action potential for the perfect performance of memory formation by LTP. Nevertheless, in the excitotoxicity condition, the overstimulation of NMDARs due to the high amount of glutamate leads to neurodegeneration. Therefore, the results of the current study suggest that the functions of neurons rely on presynaptic VGCCs. Based on the significant difference between the control and NMDA + presynaptic VGCC blocker groups, the modulation of presynaptic VGCCs failed to prevent and improve cognitive impairment completely.

LTP is a process during which neurons synaptically fire, leading to memory formation (Levenson and Sweatt, 2005). Therefore, if the neurons are healthy and active, we can observe their function. Thus, the electrophysiological information of hippocampus neurons in the mossy fiber and Schaffer collateral pathways were recorded to evaluate the episodic memory in experimental groups.

Episodic and spatial memories depend on LTP (Loprinzi et al., 2018), which in turn relies on synaptic activity (Nicoll, 2017). The synaptic activities in LTP are categorized as an enhancement of the postsynaptic response to glutamate, an increase in glutamate release from the presynaptic neurons, and the last category, which involves both activities. LTP in mossy fibers is non-associative. In other words, the activity of postsynaptic neurons is not necessary for this form of LTP in this circuit. LTP is predominately presynaptic and is stimulated by calcium influx into the neurons, which in turn, increases cyclic adenosine monophosphate (cAMP) (Alkadhi, 2021). This activates calcium/calmodulin-dependent adenylyl cyclase, which in turn activates protein kinase A. These events lead to the phosphorylation of SYN, which increases the release of excitatory neurotransmitters and as a result enhances the fEPSP (Ji et al., 2017). The results of the current study indicated that NMDA intra-hippocampal injection decreases fEPSP following LTP induction because of the destructive excitotoxicity effect. Nevertheless, the administration of presynaptic VGCC blockers restored the fEPSP to lower levels than normal through the modulation of the presynaptic VGCCs and prevention of excitotoxicity induction.

LTP form in the Schaffer collateral pathway is associative, which means that the enhancement of glutamate release from both of the presynaptic neurons and postsynaptic at the same time is required for the induction of LTP. For this purpose, CA_1_ and CA_3_ neurons must be regulated with each other. There is a retrograde pathway from CA_1_ to CA_3_ neurons in the rat hippocampus (Vadakkan, 2019). In addition, the neurons in the CA_3_ area are highly connected and have self-excitation ability (Morgado Bernal and Segura Torres, 2022). Therefore, a single administration of NMDA can trigger this self-excitation, which leads to excitotoxicity in this area. Moreover, this excitation transmits to CA_1_ neurons through the Schaffer collateral circuit, which causes more excitation through the retrograde pathway. The fEPSP amplitudes follow a reduction in LTP induction. Our data proposed that this overexcitation can be modulated in this situation by adding presynaptic VGCC blockers. Therefore, in this step, the fEPSP amplitude after LTP induction was restored nearly to a normal level, and spatial memory performance reverted to a good level compared with the NMDA-treated group.

As mentioned previously, the administration of NMDA degraded SYN expression due to its excitotoxicity effect, and the injection of presynaptic VGCC blockers increased SYN protein expression. Our findings demonstrated that the reduction and loss of SYN are higher in CA_1_ than in CA_3_ and more in CA_3_ than in the DG area (heat map of localizing SYN). In addition, this pattern of expression relies on the pathways in the hippocampus. The self-excited pyramidal neurons of CA_3_ connect to CA_1_ neurons. Therefore, the overstimulation of CA_3_ neurons is transmitted to CA_1_, and then, CA_1_ pyramidal neurons, in turn, send the signal to CA_3_ neurons through a retrograde pathway (Diana and Marty, 2004). Thereby, the excitation in the CA_1_ and CA_3_ areas will be amplified due to over-stimulation of pyramidal neurons in both regions and their connections with each other. This sequence lead to more elimination of these neurons. Hence, the excitation of mossy fiber is mainly presynaptic (Alkadhi, 2021), and postsynaptic neurons cannot influence presynaptic neurons. The loss of SYN is far less than with the Schaffer collateral circuit. In addition, there is no interaction between neurons of the DG area in excitotoxicity conditions (like CA_3_), i.e., these neurons lack self-excitation ability. Healthy pyramidal cells have a normal shape (circular), size, and membrane. Despite this, the dead pyramidal cells exhibited fragmented nuclei, membrane budding, and shrinkage.

Our results are in agreement with the previous articles published regarding presynaptic VGCC blockers in memory elimination. In our previously published studies, each small protein was used solely. Omega-lycotoxin-Gsp2671e and omega-agatoxin-Aa4b as P/Q-type VGCC blockers were used to prevent glutamate-induced excitotoxicity in the CA_3_ area (Keimasi et al., 2022; Keimasi et al., 2023). In addition, omega-agatoxin-Aa2a as an N-type VGCC blocker was used to prevent glutamate-induced excitotoxicity in the CA_3_ area of rat hippocampus (Keimasi et al., 2023a). In these studies, ameliorative effects of state-dependent small proteins versus glutamate-induced excitotoxicity were observed. In the current study, N- and P/Q-type VGCC blockers were used simultaneously in the trisynaptic pathway (DG, CA3, and CA1 areas) as a co-treatment in rat hippocampus. When N- and P/Q-type VGCC blockers were used together, the ameliorative effect against glutamate-induced excitotoxicity was significantly higher. Therefore, the concurrent use of N- and P/Q-type VGCC blockers shows a synergic effect. Additionally, our results are supported by some previously published studies in which NMDA antagonists were used against excitotoxicity (Rodrigues et al., 2001; Fontana et al., 2003; Rodrigues et al., 2004; Primini et al., 2019). In some other studies, the Parawixin protein family extracted from the *Parawixia bistriata* spider was examined. Some of these family proteins (Parawixin1, Parawixin2, and Parawixin10) are NMDA antagonists with neuroprotective effects in both *in vitro* and *in vivo* models of excitotoxicity (Fontana et al., 2007; Fachim et al., 2015; Liberato et al., 2018).

## Conclusion

Excitotoxicity, which is induced by NMDA injection into the rat hippocampus, leads to the death of pyramidal neurons in the CA_1_ and CA_3_ areas, a decrease in fEPSP after LTP induction, and the degradation of SYN protein. By contrast, the administration of omega-Lsp-IA and omega-agatoxin-Aa2a (co-treatment) as blockers of presynaptic VGCCs in NMDA-treated rats leads to the inhibition of neuronal cell death and prevention of pyramidal cell elimination, which enhances the fEPSP after LTP induction due to the modulation of presynaptic VGCCs, as well as the increase in the amount of SYN protein. The current results demonstrated that omega-Lsp-IA and omega-agatoxin-Aa2a have a restorative effect on excitotoxicity-induced memory impairment and avoid diminishing the neurons in the trisynaptic circuit of the hippocampus area. The current research is complementary to previous studies and revealed that presynaptic calcium channel (N and P/Q types) blockage in the channelopathy, due to excitotoxicity, can be very effective. Channelopathy occurs in AD and it is necessary to reduce or stop it, to inhibit AD progression in patients. The evaluation of blockers (small proteins) can be considered for future research due to their enhancement effect on L-type calcium channel expression during the aging process.

## Data Availability

The original contributions presented in the study are included in the article/supplementary materials, further inquiries can be directed to the corresponding author/s.
